# Percutaneous vertebroplasty in Moroccan patients with vertebral compression fractures

**DOI:** 10.11604/pamj.2017.26.225.9872

**Published:** 2017-04-25

**Authors:** Mustapha Hemama, Nizar El Fatemi, Rachid Gana

**Affiliations:** 1 UFR d’'Epidémiologie Clinique et Sciences Médico-Chirurgicale, Ertna-Mohammed V University- Souissi (UM5S)-Faculty of Medicine and Pharmacy, Rabat, Morocco; 2 Department of Neurosurgery, Ibn Sina University Hospital, Rabat, Morocco

**Keywords:** Vertebroplasty, vertebral compression fractures, pain

## Abstract

Incidence of vertebral compression fractures (VCFs) is increasing due to the increase in human life expectancy and prevalence of osteoporosis. Percutaneous vertebroplasty (PVP) has become a widely used alternative treatment for symptomatic VCFs refractory to medical therapy. It is a minimally invasive technique involving injection most frequently of polymethylmethacrylate (PMMA) directly into the vertebral body through the pedicles. Percutaneous vertebroplasty yields good results in terms of early vertebral stabilization and prompt pain relief. This study describes the experience of the Department of Neurosurgery at Ibn Sina University Hospital (Rabat, Morocco) and assesses short and long term clinical and radiological outcomes and complications of vertebroplasty in a retrospective assessment of 36 vertebral compression fractures in 35 patients (16 men and 19 women subjects) who had been treated with vertebroplasty from November 2006 to December 2014.

## Introduction

Percutaneous vertebroplasty (Vertebroplasty, PVP) has become a widely accepted as a safe and effective minimally invasive procedure for the treatment of painful vertebral body compression fractures (VCFs) refractory to medical therapy. Vertebroplasty is a minimally invasive image-guided procedure involving the injection of bone cement (mostly polymethylmethacrylate (PMMA)) into a vertebral body fracture in an effort to improve pain and stability of the fracture [1]. Percutaneous vertebroplasty was performed for the first time in 1984 in France by Galibert and Deramond for the treatment of an aggressive hemangioma at the C2 vertebra with very satisfying results. The proven efficacy of the procedure led to an extension of its indications to metastatic and myelomatous osteolytic lesions, and then to osteoporotic vertebral compression fractures (VCFs). The application of vertebroplasty to osteoporotic VCF was first published in 1989 [1]. Over the last years, osteoporotic fractures have become the main indication for vertebroplasty [2]. The purpose of this retrospective observational study, which represents the largest Moroccan study on vertebroplasty to-date, is to report the experience of the Department of Neurosurgery at Ibn Sina University Hospital (Rabat, Morocco) and assess the effectiveness of the technique in achieving pain relief and improvement in function in patients suffering from medically refractory VCFs.

## Methods

We retrospectively studied the charts of 35 patients (16 men and 19 women subjects) hospitalized for VCFs in the Department of Neurosurgery at Ibn Sina University Hospital from November 2006 to December 2014 and who had undergone percutaneous vertebroplasty. The decision of vertebroplasty was previously systematically discussed in the context of a multidisciplinary meeting attended by neurosurgeons and radiologists. Patients had to have one or more severe vertebral fractures and be resistant to medical treatment. None of the patients presented the classic contraindications to vertebroplasty: coagulopathies, rupture of the posterior wall or cement allergy. Before the procedure, all the aspects were explained to the patients and detailed biochemical, radiological and neurological evaluations were performed including pain assessment with the use of the Visual Analogue Scale (VAS). A pre-anesthetic consultation was also carried out. The procedure was performed under general anesthesia or under sedation and local anesthesia at the injection site. An antibioprophylaxis was performed systematically before the beginning of the procedure. The injection of PMMA was done under permanent fluoroscopic control in a profile position to verify the absence of posterior leakage of cement to the spinal canal. The volume of cement injected was determined by the operator intraoperatively as that required to adequately fill the vertebral body. Postoperative evaluation included a full clinical assessment and, when appropriate, a radiological investigation with plain radiographs, CT and MR imaging. The mean follow-up period was 12.1±3.6 (range: 6-42 months). Data were analyzed using SPSS (IBM SPSS statistics 20.0). All measurement data were presented as mean ± SD. Pre-and post-operative data were compared using a student's t-test. A p value <0.05 was considered as statistical difference.

## Results

Thirty five (35) subjects, 16 (45.7%) men and 19 women (54.3%), mean age 56 ± 11 years and all having at least one vertebral body compression fracture have benefited from vertebroplasty Table 1. A total of 36 VCFs were observed (one patient had a VCF at two different vertebrae). Treated VCFs were mostly lumbar (70%) and dorsal in 30% of the cases. The fractures were mostly due to post-traumatic fractures (36.1%) or from osteoporotic origin (33.3% of the cases). Vertebral compression fractures were osteopenic in 6 cases (16.7%), neoplastic in 3 (8.3%) and due to Kahler's disease in 2 patients (5.6% of the cases). In 33 patients, the mobility was recovered in less than 24 hours without the need of a bracet (corset) or orthosis. The two other patients recovered mobility between 24 and 36 hours with a rapid return to physical activity. Before the procedure, all the patients suffered disabling pain with a mean VAS score of 8.0 ± 0.8. Twenty four (24) hours after the procedure, all the patients declared an important pain relief with a mean VAS score of 1.6 ± 0.7. Pain intensity was significantly different pre- and post-procedure (p<0.05). The kyphosis decreased by a mean of 4.89° after vertebroplasty. In the long term (mean follow-up period was 12.1 ± 3.6 (range: 6-42 months)), 33 patients declared being relieved in relation to the pain they experienced before the procedure (VAS score of 1.7 ± 0.5). Two patients have residual pain remotely from the treated vertebra with a VAS score of 4. None of the patients declared a loss of autonomy or a decrease in his activity. There was no return of the tumoural activity in the 2 patients with Kahler's disease. One patient had cement embolism and another subject had cement leak. Finally, in 1 patient, a neurological deterioration in the follow-up period was observed and required a decompressive laminectomy.

**Table 1:** Demographic and clinical characteristics of study population

Study Population	
Age	
Mean ± SD [range]	56 ± 11 years [21-75] years]
**Gender (N (%))**	
Men	16 (45.7%)
Women	19 (54,3%)
**Vertebra (N (%))**	
D11	3 (8.3%)
D12	3 (8.3%)
L1	18 (50%)
L2	6 (16.7%)
L3	3 (8.3%)
L4	3 (8.3%)
**Etiology of the VCF (N (%))**	
Osteoporotic	12 (33.3%)
Osteopenic	6 (16.7%)
Neoplastic	3 (8.3%)
Post-traumatic	13 (36.1%)
Kahler’s disease	2 (5.6%)

## Discussion

Percutaneous vertebroplasty (PVP) is an accepted treatment modality for osteoporotic, malignant, and traumatic spinal fractures. The effectiveness of this technique has been demonstrated in numerous studies [3-6]. However, the efficacy of PVP has been questioned in different randomized clinical trials (RCTs). Buchbinder et al, (2009) [7] published a randomized multicenter controlled trial where 78 patients were randomized to vertebroplasty or sham. No difference in pain (overall, at night, at rest) and quality of life was found at 1 week or at 1, 3, or 6 months after treatment. Kallmes et al, (2009) [8] randomized 131 patients to vertebroplasty versus sham. Both groups had immediate improvement in disability and pain scores after the intervention. Although the two groups did not differ significantly on any secondary outcome measure at 1 month, there was a trend toward a higher rate of clinically meaningful improvement in pain in the vertebroplasty group. However, these two studies were heavily criticized because of the inclusion of patients with subacute and chronic fractures and so should be interpreted cautiously [9, 10]. Since then, seven RCT have been published, with positive results in six of them. The exception was a comparison of 49 patients with acute/semiacute OVF treated conservatively or with percutaneous vertebroplasty; an immediate reduction in pain was observed in the vertebroplasty group, but the results between the groups were similar at 3 and 12 months [11]. In our study, the results clearly show that PVP provides an early relief (since Day 1) for VCFs from different origins. Pain intensity was significantly different pre- and post-procedure (p<0.05) and the mobility was recovered in nearly all the patients in the 24 hours following the procedure. The kyphosis decreased by a mean of 4.89° after vertebroplasty. These effects were significant and maintained also at the final assessment.

The advantage of vertebroplasty in malignant spine disease is the less invasive nature compared to open spinal surgery and the apparent rapid pain relief compared to radiotherapy and other conventional treatment options. In the present study, the patients with painful spine metastasis were successfully treated without serious complications. The mechanism of pain relief from PVP remains uncertain. The stabilization of microfractures, as well as vascular, chemical, and thermal factors, have been proposed as mechanisms. Pain receptors are destroyed by an exothermic reaction of the cement and also due to the compression of small nerve endings [12]. Absolute contraindications of PVP include treatment of asymptomatic VCFs and treatment of patients improving with conservative medical care. Prophylactic treatment in osteoporotic patients without a VCF is not viewed as an acceptable indication. Additional absolute contraindications include uncorrectable coagulopathy, and active local or systemic infection. Allergy to PMMA or other bone cement products preclude PVP. Relative contraindications include disruption of the posterior vertebral body wall or tumor extension into the spinal canal. The treatment of very severely compressed VCF, defined as vertebral body collapse to less than one-third of the original height, is also considered a relative contraindication. Treatment of these fractures is more technically challenging and is often associated with increased rates of complications [2]. Potential complications of PVP procedures include pulmonary embolism, cardiac perforation, epidural cement extravasation causing spinal cord or nerve root compression, cement leak into paraspinal, intradiskal or venous systems, infections, and fractures in adjacent vertebrae [13]. There were no major complications in our study cohort, except for one patient who had cement embolism, another subject who had cement leak and a neurological deterioration in one patient in the follow-up period that required a decompressive laminectomy. These results indicate that PVP is generally a safe procedure for patients with VCFs. Our study is limited by its sample size and the selection and review biases inherent in many retrospective observational studies. The accuracy and uniformity of data gathered retrospectively often are poorer than for data gained prospectively. We report statistically significant improvements in pain and mobility but we had no control group for comparison. We cannot know the extent to which factors such as the placebo effect, regression to the mean, and natural history of the disease may have biased conclusions made from these data.

## Conclusion

PVP prevents immobility in patients with vertebral compression fractures from different origins. It is a safe surgical option with minimal complications and it decreases spinal deformity, restored sagittal alignment and prevents its progression caused by fracture. This procedure can be performed rapidly and is an alternative to open surgery in patients with comorbidities.

### What is known about this topic

- Vertebroplasty is a non-invasive surgical procedure to treat vertebral compression fractures;
- Vertebroplasty reduces pain and improves function;
- Vertebroplasty improves quality of life.

### What this study adds

- First Moroccan study on vertebroplasty;
- Largest Moroccan study on vertebroplasty to-date;
- Confirmation of the efficacy of vertebropasty in VCFs from different origins.
